# Physical activity associations with physical function and body composition among community‐dwelling older adults in Japan: The Kyotango Longevity Cohort Study

**DOI:** 10.1111/ggi.70189

**Published:** 2025-09-29

**Authors:** Motonori Kubo, Norikazu Hishikawa, Hironari Shinjo, Suzuyo Ohashi, Koshiro Sawada, Satoaki Matoba, Yasuo Mikami

**Affiliations:** ^1^ Department of Rehabilitation Medicine, Graduate School of Medical Science Kyoto Prefectural University of Medicine Kyoto Japan; ^2^ Department of Development of Multidisciplinary Promote for Physical Activity Kyoto Prefectural University of Medicine Kyoto Japan; ^3^ Department of Rehabilitation, University Hospital Kyoto Prefectural University of Medicine Kyoto Japan; ^4^ Department of Longevity and Regional Epidemiology Kyoto Prefectural University of Medicine Kyoto Japan

**Keywords:** body composition, healthy longevity, older adult, physical activity, physical function

## Abstract

**Aim:**

Physical activity benefits health, whereas physical inactivity increases the risk of age‐related conditions and adverse health outcomes. Kyotango City in Japan is known for its residents' longevity, and many centenarians live there. This study investigated the physical activity associations with physical function and body composition in older adults living in this area.

**Methods:**

Community‐dwelling older adults (*n* = 727; mean age 74.1 years; 59.4% women) participated in this study. Multiple linear regression analyses were carried out to examine objectively‐measured physical activity associations with physical function (handgrip strength, knee extension strength and maximum gait speed) and body composition (skeletal muscle mass index and whole‐body phase angle), adjusting for potential confounders. Additionally, isotemporal substitution models were applied to estimate changes resulting from replacing sedentary behavior with light‐intensity physical activity (LPA) and moderate‐to‐vigorous‐intensity physical activity (MVPA).

**Results:**

All physical function and body composition indicators were significantly associated with SB and MVPA (all *P* < 0.050). However, LPA was associated with only handgrip strength and body composition indicators. Replacing 30 min of SB per day with LPA was associated with a 0.176 kg increase in handgrip strength, 0.018 kg/m^2^ increase in skeletal muscle mass index and 0.016° increase in whole‐body phase angle. Alternatively, replacing sedentary behavior with MVPA improved all physical function and body composition indicators.

**Conclusions:**

Older adults in a region of Japan known for longevity might maintain physical function and favorable body composition through engaging in abundant physical activity. Public health strategies should prioritize MVPA promotion while recognizing LPA's complementary role for physically inactive older adults. **Geriatr Gerontol Int 2025; 25: 1511–1517**.

## Graphical abstract

Among older adults living in Kyotango City, a longevity region of Japan, replacing sedentary behavior primarily with moderate‐to‐vigorous‐intensity physical activity was positively associated with muscle strength, gait speed, skeletal muscle mass and phase angle. These findings support promoting physical activity to help maintain physical function and body composition.

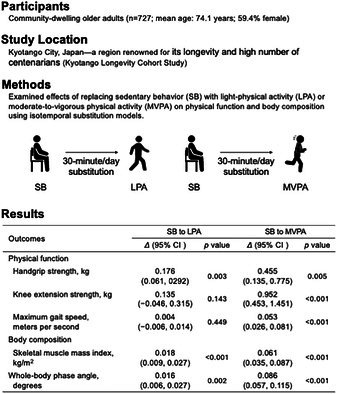

## Introduction

Healthy longevity is a universal goal for humanity, as reflected in the United Nations Sustainable Development Goal 3, which aims to ensure healthy lives and promote well‐being.^1^ However, aging is a progressive and irreversible process that inevitably deteriorates body structures and functions. With the rapid aging of the population globally, substantial burdens on individuals, families and society are on the rise, as many older adults develop age‐related declines in physical function and adverse changes in body composition.^2^, ^3^, ^4^ Physical activity, shaped by daily living patterns, is a key factor contributing to healthy longevity.^5^ Physical activity encompasses any bodily movement produced by skeletal muscles that results in energy expenditure.^6^ This includes all forms of movement, such as leisure time physical activity, active transportation, occupational tasks and domestic activities. Conversely, physical inactivity increases the risk of non‐communicable diseases and other adverse health outcomes.^7^ Recent estimates suggest that insufficient physical activity causes 5 million deaths annually, resulting in approximately US$67.5 billion in healthcare expenses globally.^8^ This underscores the widespread nature of physical inactivity among older adults. Therefore, promoting physical activity and preventing deterioration in physical function and body composition are crucial for supporting healthy longevity among older populations.

Japan's aging rate (the ratio of persons aged ≥65 years to the total population) reached 25% in 2013, the highest in the world. It is projected to exceed 30% in 2025 and 39.9% in 2060.^9^ According to the World Health Statistics 2023, average life expectancy is 73.3 years globally, but 84.3 years in Japan—the highest among all World Health Organization member states.^10^ Kyotango City (Japan) has a population of approximately 50 500, and 38.0% of residents are aged ≥65 years, making it one of the most aged areas in Kyoto Prefecture. The city is distinguished by its high longevity, with 114 centenarians—equivalent to 226 per 100 000 residents—approximately threefold the national average and 2.7‐fold the Kyoto Prefecture average, highlighting it as one of Japan's notable regions for longevity. The Kyotango Longevity Cohort Study, which focuses on this region, has previously investigated various factors regarding healthy longevity, including gut microbiota and oral function.^11^, ^12^, ^13^ However, physical activity, physical function and body composition associations with longevity in this region have not been studied.

This study aimed to examine the associations among physical activity, physical function and body composition in community‐dwelling older adults, and to explore the potential benefits of increasing physical activity on these health‐related factors, using data from the ongoing Kyotango Longevity Cohort Study.

## Methods

### Study design and ethics

A cross‐sectional analysis of data from the Kyotango Longevity Cohort Study, initiated by the Department of Longevity and Community Epidemiology at Kyoto Prefectural University of Medicine (Kyoto, Japan), was carried out. The affiliated institution's Ethics Review Board approved the study protocol (approval number: ERB‐C‐885‐8). This study was carried out in accordance with the Code of Ethics of the World Medical Association (Declaration of Helsinki). Written informed consent was obtained from all participants.

### Participants

Community‐dwelling older adults (aged ≥65 years) were recruited from Kyotango City between August 2017 and September 2024. Study recruitment was promoted, as part of the longevity health checkup program, through the website and public relations magazine of Kyotango City Yasaka Hospital (Kyotango, Japan). Participants completed a questionnaire regarding their demographic information. Additionally, anthropometric measurements, physical function and body composition assessments, and objective physical activity monitoring were carried out.

### Data collection

Demographic data—including age, sex, smoking status, drinking status, marital status, educational attainment, and history of hypertension, diabetes, dyslipidemia, cardiovascular disease and stroke—were collected using a self‐administered questionnaire. In addition, anthropometric measurements, including height, weight and body mass index, were individually measured.

### Physical activity monitoring

Physical activity was measured using 45 triaxial accelerometers (Actigraph GT3X+; ActiGraph LLC, Fort Walton Beach, FL, USA). At their health checkup visits, medical staff explained the procedures and distributed the devices to participants. Participants wore an accelerometer on an elastic belt over the right side of the waist at all times for 7 consecutive days, except during water‐based activities (e.g. bathing or swimming). The device recorded data at 30 Hz and was returned by mail after the monitoring period. Sleep periods were excluded based on participants' self‐reported sleep diaries. If sleep diary data were unavailable, standard bedtime (23:00 hours) and wake‐up time (06:00 hours) were applied. A valid monitoring period comprised at least 4 days of wear, including at least one weekend day. After monitoring, the data were aggregated into 60‐s epochs. Non‐wear periods (≥90 min of consecutive zero counts or nonzero counts, with an allowance of 2‐min intervals of nonzero counts with an upstream or downstream 30‐min consecutive zero‐count window for detection of artifactual movements) were excluded from each participant's data file.^14^, ^15^ Only data from participants who wore the device for ≥10 h per day on at least 4 days were included in the final analysis.

The metabolic equivalent of a task (MET) is a physiological measure that quantifies the energy expenditure of an activity relative to resting metabolism. In general, physical activities can be classified according to their MET values: sedentary behavior (SB) is defined as ≤1.5 METs (e.g. watching TV), light‐intensity physical activity (LPA) is 1.6–2.9 METs (e.g. light household chores) and moderate‐to‐vigorous‐intensity physical activity (MVPA) is ≥3 METs (e.g. walking, gardening or stair climbing).^16^ Physical activity intensity in this study was classified based on vector magnitude counts using the following cut‐points: SB, < 200 counts/min; LPA, 200–2689 counts/min; and MVPA, ≥2690 counts/min.^17^, ^18^ Data processing was carried out using Actilife® version 6.13.4 (ActiGraph, Fort Walton Beach, FL, USA), with the low‐frequency filter applied to enhance sensitivity to low‐intensity activities.^19^

### Physical function and body composition assessments

Physical function was assessed using three indicators: handgrip strength, knee extensor strength and maximum gait speed. Handgrip strength was measured using a Jamar hydraulic hand dynamometer (SH5001; SAKAI Medical, Tokyo, Japan), with the maximum value recorded from two trials on each hand. Knee extensor strength was measured using a hand‐held dynamometer (μTas F‐1; Anima, Tokyo, Japan) while the participant carried out a maximal‐effort isometric knee extension in a sitting position. The higher value of two measurements on the right leg was recorded. Maximum gait speed was evaluated on a 16‐m long overground walkway. Participants were instructed to walk at their fastest pace, and the time to traverse the central 10‐m section was measured using a stopwatch and used to calculate gait speed. Body composition was assessed using the skeletal muscle mass index and whole‐body phase angle. Both were measured via bioelectrical impedance analysis using a body composition analyzer (InBody 770; InBody, Seoul, South Korea). The skeletal muscle mass index was calculated by dividing the total lean muscle mass of the four extremities by height squared. Whole‐body phase angle was calculated as the arctangent of reactance to resistance and expressed in degrees. Phase angle is commonly used to evaluate cellular health, nutritional status and muscle quality, with higher values indicating better cellular integrity and function.^20^

### Statistical analysis

Demographic, physical activity, physical function and body composition data were summarized as means and standard deviations or counts and percentages. Age was categorized into five groups: 65–69, 70–74, 75–79, 80–84 and ≥85 years, and summarized as counts and percentages. Student's *t*‐tests, χ^2^‐tests or Mann–Whitney *U*‐tests were used to compare these parameters between sexes. When significant differences in age or total daily accelerometer wear time were observed between sexes, an analysis of covariance (ANCOVA) was carried out to compare physical activity, physical function and body composition between men and women. For physical activity comparisons, both age and total daily accelerometer wear time were included as covariates, whereas only age was included as a covariate for comparisons of physical function and body composition. These data are presented as estimated marginal means (EMMs) with standard errors.

Multiple linear regressions based on a single‐factor model were carried out to examine whether physical function and body composition parameters were independently associated with physical activity, adjusting for potential confounders. In addition, multiple linear regression based on an isotemporal substitution model was carried out to estimate the effect of replacing 30 min of SB per day with physical activity of varying intensities on physical function and body composition.^21^ Potential confounding factors included sex, age, height, weight, smoking status, drinking status, marital status, educational attainment, hypertension, diabetes, cardiovascular disease, stroke and total daily accelerometer wear time. Multicollinearity was assessed using the variance inflation factor, with values between 1 and 10 indicating its absence. All statistical analyses were carried out using IBM SPSS Statistics for Windows, version 29.0 (IBM, Armonk, NY, USA), and statistical significance was set at *P* < 0.05.

## Results

**Figure 1:**
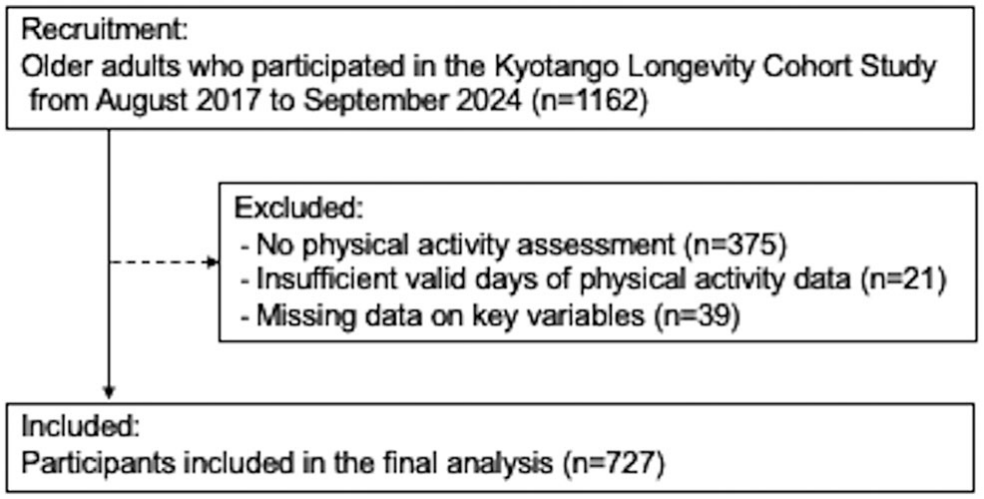
Flow diagram of the participant selection process. The exclusion criteria applied during participant recruitment and the final sample included in the analysis are shown.

A total of 727 participants (295 [40.6%] men and 432 [59.4%] women) were included in the final analysis (Fig. 1). Tables 1 and 2 summarize the participants' demographic, anthropometric, physical activity, physical function, and body composition data overall and stratified by sex. The mean age of all participants was 74.1 years, with men being significantly older than women (75.1 years vs 73.3 years, *P* < 0.001). Men also had significantly greater height, weight and body mass index than women (all *P* < 0.001).

**Table 1:** Demographic characteristics and anthropometric measurements

	Overall (*n* = 727)	Men (*n* = 295)	Women (*n* = 432)	*P*‐value
Age, years	74.1 (5.5)	75.1 (5.8)	73.3 (5.2)	<0.001
Age group, *n* (years)				
65–69 70–74 75–79 80–84 ≥85	186 [25.6] 256 [35.2] 177 [24.3] 84 [11.6] 24 [3.3]	59 [20.0] 99 [33.6] 79 [26.8] 42 [14.2] 16 [5.4]	127 [29.4] 157 [36.3] 98 [22.7] 42 [9.7] 8 [1.9]	0.002
Height (cm)	157.4 (8.4)	164.6 (6.4)	152.4 (5.5)	<0.001
Weight (kg)	56.6 (9.9)	63.5 (9.2)	51.8 (7.3)	<0.001
BMI (kg/m^2^)	22.8 (3.0)	23.4 (2.9)	22.3 (3.0)	<0.001
Smoking (*n*)				
Current smoker Never smoked or a past smoker	27 [3.7] 700 [96.3]	22 [7.5] 273 [92.5]	5 [1.2] 427 [98.8]	<0.001
Drinking (*n*)				
Current drinker Non‐drinker or a past drinker	278 [38.2] 449 [61.8]	181 [61.4] 114 [38.6]	97 [22.5] 335 [77.5]	<0.001
Marital status (*n*)				
Married Not married	550 [75.6] 177 [24.4]	266 [90.2] 29 [9.8]	284 [65.7] 148 [34.3]	<0.001
Educational attainment (*n*)				
Bachelor's degree Less than a bachelor's degree	112 [15.4] 615 [84.6]	84 [28.5] 211 [71.5]	28 [6.5] 404 [93.5]	<0.001
Hypertension (*n*)				
Under treatment Not under treatment	263 [36.1] 465 [63.9]	126 [42.7] 169 [57.3]	137 [31.7] 295 [68.3]	<0.001
Diabetes (*n*)				
Under treatment Not under treatment	84 [11.5] 644 [88.5]	48 [16.3] 247 [83.7]	36 [8.3] 396 [91.7]	<0.001
Dyslipidemia (*n*)				
Under treatment Not under treatment	222 [30.5] 505 [69.5]	64 [21.7] 231 [78.3]	158 [36.6] 274 [63.4]	<0.001
Cardiovascular disease (*n*)				
Under treatment Not under treatment	49 [6.7] 678 [93.3]	33 [11.2] 262 [88.8]	16 [3.7] 416 [96.3]	<0.001
Stroke (*n*)				
Under treatment Not under treatment	14 [1.9] 713 [98.1]	8 [2.7] 287 [97.3]	6 [1.4] 426 [98.6]	0.200

Data are presented as the mean (standard deviation) or count [percentage]. Statistical analyses were carried out using a Student's *t*‐test or a χ^2^‐test. BMI, body mass index.

**Table 2:** Physical activity, physical function and body composition

	Overall (*n* = 727)	Men^†^ (*n* = 295)	Women^†^ (*n* = 432)	*P*‐value
Physical activity				
SB (min/day)	466.2 (113.1)	503.0 (6.2)	441.1 (5.1)	<0.001
LPA (min/day)	459.8 (108.6)	416.3 (5.5)	489.6 (4.5)	<0.001
MVPA (min/day)	45.6 (34.5)	52.4 (2.0)	40.9 (1.6)	<0.001
Physical function				
Handgrip strength (kg)	29.3 (8.4)	34.5 (0.3)	23.8 (0.3)	<0.001
Knee extension strength (kg)	28.7 (9.2)	35.1 (0.4)	24.4 (0.4)	<0.001
Maximum gait speed (m/s)	2.2 (0.5)	2.3 (0.02)	2.1 (0.02)	<0.001
Body composition				
Skeletal muscle mass index (kg/m^2^)	6.5 (1.0)	7.4 (0.04)	5.9 (0.04)	<0.001
Whole‐body phase angle (degrees)	4.5 (0.6)	5.0 (0.03)	4.3 (0.02)	<0.001

Statistical analyses were carried out using a Student's *t*‐test or Mann–Whitney *U*‐test. The overall data are presented as the mean (standard deviation). ^†^Estimated marginal means (standard errors) were calculated for men and women. Age and total daily accelerometer wear time were included as covariates for physical activity, whereas age alone was included as a covariate for physical function and body composition. LPA, light‐intensity physical activity; MVPA, moderate‐to‐vigorous‐intensity physical activity; SB, sedentary behavior.

A significantly higher proportion of men were current or past smokers and drinkers, were married, and had attained a bachelor's degree or higher (all *P* < 0.001). Men had higher rates of hypertension, diabetes and cardiovascular disease (all *P* < 0.001), whereas women had a higher rate of dyslipidemia (*P* < 0.001). The overall durations of SB, LPA and MVPA, as well as the estimated marginal means and standard errors of physical activity by sex, adjusted for age and total daily accelerometer wear time, are summarized in Table 2. A significant sex difference was observed in total daily accelerometer wear time, with men wearing it for 959.9 min per day and women for 979.6 min per day (*P* < 0.001). Significant differences were observed between sexes for all physical activity intensities, adjusted for age and total daily accelerometer wear time (all *P* < 0.001). After adjustment for age, the estimated marginal means for physical function and body composition showed that men had significantly higher handgrip strength, knee extension strength, maximum gait speed, skeletal muscle mass index and whole‐body phase angle compared with women (all *P* < 0.001).

After adjusting for potential confounders, all physical function and body composition indicators were significantly associated with SB and MVPA (all *P* < 0.001; Table 3). Although LPA was significantly associated with only some physical function indicators, it showed significant associations with all body composition indicators (*P* < 0.001). Replacing 30 min of SB per day with an equivalent amount of LPA led to partial increases in physical function measures and increases in all body composition measures (Table 4). In contrast, replacing SB with MVPA increased all measures of physical function and body composition. All variance inflation factor values were between 1 and 10, indicating no multicollinearity.

**Table 3:** Associations of physical activity with physical function and body composition

	SB				LPA				MVPA			
	*β*	95% CI		*P*‐value	*β*	95% CI		*P*‐value	*β*	95% CI		*P*‐value
		Lower	Upper			Lower	Upper			Lower	Upper	
Physical function												
Handgrip strength	−0.098	−0.144	−0.052	<0.001	0.087	0.037	0.136	<0.001	0.072	0.029	0.116	0.001
Knee extension strength	−0.105	−0.170	−0.039	0.002	0.073	0.003	0.144	0.042	0.126	0.064	0.187	<0.001
Maximum gait speed	−0.093	−0.166	−0.019	0.013	0.054	−0.025	0.132	0.182	0.138	0.070	0.207	<0.001
Body composition												
Skeletal muscle mass index	−0.095	−0.127	−0.063	<0.001	0.080	0.045	0.114	<0.001	0.081	0.051	0.111	<0.001
Whole‐body phase angle	−0.169	−0.229	−0.110	<0.001	0.128	0.064	0.192	<0.001	0.180	0.125	0.236	<0.001

Statistical analysis was carried out using multiple linear regression based on a single‐factor model. *β*, standardized partial regression coefficient; CI, confidence interval; LPA, light‐intensity physical activity; MVPA, moderate‐to‐vigorous‐intensity physical activity; SB, sedentary behavior.

**Table 4:** Estimated changes in physical function and body composition after 30‐min isotemporal substitution of sedentary behavior with light‐intensity physical activity or moderate‐to‐vigorous‐intensity physical activity

	SB to LPA				SB to MVPA			
	Change in outcomes	95% CI		*P*‐value	Change in outcomes	95% CI		*P*‐value
		Lower	Upper			Lower	Upper	
Physical function								
Handgrip strength (kg)	0.176	0.061	0.292	0.003	0.455	0.135	0.775	0.005
Knee extension strength (kg)	0.135	−0.046	0.315	0.143	0.952	0.453	1.451	<0.001
Maximum gait speed (m/s)	0.004	−0.006	0.014	0.449	0.053	0.026	0.081	<0.001
Body composition								
Skeletal muscle mass index (kg/m^2^)	0.018	0.009	0.027	<0.001	0.061	0.035	0.087	<0.001
Phase angle of the whole body (degrees)	0.016	0.006	0.027	0.002	0.086	0.057	0.115	<0.001

Statistical analysis was carried out using multiple linear regression based on an isotemporal substitution model. CI, confidence interval; LPA, light‐intensity physical activity; MVPA, moderate‐to‐vigorous‐intensity physical activity; SB, sedentary behavior.

## Discussion

This study investigated physical activity associations with physical function and body composition among community‐dwelling older adults residing in a region of Japan known for longevity. After adjusting for potential confounders, multiple regression analyses showed that physical function and body composition were primarily associated with SB and MVPA. Specifically, replacing 30 min of SB per day with MVPA resulted in higher values across all indicators, whereas replacing SB with LPA yielded only limited benefits.

Maintaining physical activity is crucial for preventing declines in physical function and adverse changes in body composition—conditions characteristic of aging. Physical activity guidelines recommend older adults engage in at least 150 min of MVPA per week (approximately 20 min per day).^22^ However, few older adults meet these recommendations.^23^ Unlike many previous studies relying on self‐reported data, this study objectively measured MVPA using triaxial accelerometers, thereby minimizing recall bias and overestimating physical activity.^24^ Previous studies using triaxial accelerometers have shown that engaging in more than 43.3 min, 28.0 min or 15.0–20.0 min of MVPA per day is associated with reduced risk of frailty, locomotor syndrome and sarcopenia, respectively.^25^, ^26^, ^27^ Overall, participants in this study averaged 45.6 min of MVPA per day, equivalent to approximately 319 min per week, more than double the recommended 150 min per week. This finding might reflect the active lifestyles of our participants residing in a region known for longevity. Kyotango City is a rural, coastal area >120 km from Kyoto City. It is characterized by mountainous terrain, a rich natural environment and a high proportion of older adults engaged in traditional lifestyles, including farming. Older adults residing in rural areas of Japan often engage in more MVPA than their urban counterparts.^28^ In addition to regional differences, environmental and lifestyle factors characteristic of rural living might further promote physical activity. In Japan, older adults residing in hilly areas are more likely to meet physical activity guidelines, and access to natural surroundings and aesthetically pleasant landscapes is associated with higher MVPA engagement.^29^, ^30^ Furthermore, frequent participation in agricultural activities is associated with better management of chronic diseases, suggesting that such tasks might also serve as a natural form of physical exertion.^31^ Taken together, these findings suggest that geographical and lifestyle factors—terrain, access to nature and agricultural practices—might contribute to the elevated MVPA observed among older adults living in rural longevity regions, such as Kyotango City.

Several sex differences were evident in this study. Men showed higher physical function and body composition outcomes than women, consistent with previous findings.^32^, ^33^ Men spent more time in SB and MVPA, and less time in LPA compared with women. Traditional gender roles in Japan might partly explain these differences; men are more likely to engage in outdoor activities, whereas women are more involved in household chores.^34^, ^35^ However, regarding differences in physical activity, the question of why women tend to achieve greater healthy life expectancy than men has not yet been fully addressed.^36^ When physical activity is assessed based on the current physical activity guidelines,^22^ men are generally more physically active than women across most age groups. Future research should explore these multidimensional determinants to inform tailored and sex‐specific interventions promoting healthy longevity.

This study showed significant associations between physical activity and most measures of physical function and body composition, supporting the beneficial role of physical activity in preventing age‐related changes in older adults. This study applied multiple linear regression analysis based on an isotemporal substitution model. Our simulation showed that replacing SB with LPA increased some indicators, except for outcomes requiring high‐intensity lower limb muscle output, such as knee extension strength and maximum gait speed. In contrast, replacing SB with MVPA resulted in significantly higher values across all indicators. These findings underscore the importance of MVPA in enhancing physical function and body composition, while also highlighting the limitations of LPA. Although some previous studies link increased LPA to health benefits, its effectiveness remains debated.^32^, ^37^, ^38^ Given that increasing LPA might be more feasible for older adults than increasing MVPA, clarifying the specific benefits and limitations of LPA is crucial for designing effective interventions. Our findings suggest that, although LPA might contribute to maintaining body composition, it might not provide sufficient stimulus to induce changes in physical function. Therefore, public health strategies to promote physical activity and support healthy longevity should prioritize increasing MVPA wherever feasible, while also recognizing the potential complementary role of LPA, particularly among physically inactive older adults with limited mobility or chronic conditions. These strategies might also contribute to achieving the UN's SDG 3, which aims to ensure healthy lives and promote well‐being for people of all ages.

This study had several strengths. First, physical activity was objectively measured using triaxial accelerometers, allowing for accurate SB, LPA and MVPA quantification. Second, multiple regression analysis based on an isotemporal substitution model was applied to estimate the effects of replacing SB with LPA or MVPA, providing practical implications for public health strategies targeting older adults. Finally, the large sample size from a region in Japan known for longevity enabled a robust analysis suggesting an association between physical activity and healthy longevity. However, several limitations should be noted. First, the cross‐sectional design of this study does not allow for causal inferences. Second, although triaxial accelerometers provide objective physical activity assessments, they might not fully capture non‐ambulatory movements, such as upper‐limb activity or specific household tasks. Third, potential seasonal variations in the measurement of physical activity were not accounted for in this study. Fourth, the β values for the associations of physical activity with physical function and body composition were approximately 0.1. Although statistical significance was achieved owing to our large sample size, these values represent small effect sizes, and clinical relevance should therefore be interpreted with caution. Fifth, although this analysis adjusted for major confounders, unmeasured variables, such as nutritional status, psychological factors, and sport and exercise habits, could have affected the results. In addition, because MVPA was defined as physical activity ≥3 METs, activities ranging from walking to vigorous‐intensity physical activity were grouped into one category, which might limit the interpretation of the findings. Sixth, although this study simulated a 30‐min substitution of SB, further investigation is needed to determine the most appropriate replacement duration. Finally, the study population consisted of relatively healthy and active older adults in Japan living in a region known for longevity, which might limit the generalizability of the findings to other populations. Despite these limitations, this study adds valuable evidence to the growing body of research emphasizing the role of physical activity in supporting physical function and body composition in older adults. Future studies should explore the feasibility and efficacy of implementing behavior‐specific and regionally tailored physical activity interventions that reduce SB and promote LPA or MVPA in diverse older populations.

This study shows that abundant physical activity benefits physical function and body composition among older adults living in a region of Japan known for longevity. These findings suggest that reducing SB and increasing physical activity—particularly MVPA—might be effective strategies for preventing unfavorable age‐related changes in physical function and body composition.

## Disclosure statement

The authors declare no conflict of interest.

## Author contributions

Conceptualization and methodology, MK and HS; data curation, MK and HS; validation, HS; formal analysis, HS; investigation, MK and SO; writing—original draft preparation, MK and NH; Visualization, MK and NH; writing—review and editing, KS and YM; supervision, SM; project administration, SO; funding acquisition, YM. All authors approved the final version of the manuscript.

## Ethics statement

This protocol was approved by the Ethics Review Board of the affiliated institution (approved number: ERB‐C‐885‐8).

## Patient consent statement

Informed consent was obtained from all participants involved in the study. Written informed consent was obtained from the patients to publish this paper.

## Data Availability

The data that support the findings of this study are available from the corresponding author upon reasonable request.

## References

[1] United Nations . Transforming our World: the 2030 Agenda for Sustainable Development. New York: United Nations, 2015.

[2] Auyeung TW , Lee SW , Leung J , Kwok T , Woo J . Age‐associated decline of muscle mass, grip strength and gait speed: a 4‐year longitudinal study of 3018 community‐dwelling older Chinese. Geriatr Gerontol Int 2014; 14: 76–84. 24450564 10.1111/ggi.12213

[3] Shimokata H , Ando F , Yuki A , Otsuka R . Age‐related changes in skeletal muscle mass among community‐dwelling Japanese: a 12‐year longitudinal study. Geriatr Gerontol Int 2014; 14: 85–92. 24450565 10.1111/ggi.12219

[4] Makizako H , Shimada H , Doi T *et al*. Age‐dependent changes in physical performance and body composition in community‐dwelling Japanese older adults. J Cachexia Sarcopenia Muscle 2017; 8: 607–614. 28597612 10.1002/jcsm.12197 PMC5566639

[5] Ammar A , Trabelsi K , Hermassi S *et al*. Global disease burden attributed to low physical activity in 204 countries and territories from −1990 to 2019: insights from the global burden of disease 2019 study. Biol Sport 2023; 40: 835–855. 37398951 10.5114/biolsport.2023.121322 PMC10286621

[6] Caspersen CJ , Powell KE , Christenson GM . Physical activity, exercise, and physical fitness: definitions and distinctions for health‐related research. Public Health Rep 1985; 100: 126–131. 3920711 PMC1424733

[7] Lee IM , Shiroma EJ , Lobelo F , Puska P , Blair SN , Katzmarzyk PT . Effect of physical inactivity on major non‐communicable diseases worldwide: an analysis of burden of disease and life expectancy. Lancet 2012; 380: 219–229. 22818936 10.1016/S0140-6736(12)61031-9 PMC3645500

[8] Ding D , Lawson KD , Kolbe‐Alexander TL , Finkelstein EA , Katzmarzyk PT , van Mechelen W . The economic burden of physical inactivity: a global analysis of major non‐communicable diseases. Lancet 2016; 388: 1311–1324. 27475266 10.1016/S0140-6736(16)30383-X

[9] Arai H , Ouchi Y , Toba K *et al*. Japan as the front‐runner of super‐aged societies: perspectives from medicine and medical care in Japan. Geriatr Gerontol Int 2015; 15: 673–687. 25656311 10.1111/ggi.12450

[10] World Health Organization . World Health Statistics 2023: Monitoring Health for the SDGs, Sustainable Development Goals. Geneva: World Health Organization, 2023.

[11] Naito Y , Takagi T , Inoue R *et al*. Gut microbiota differences in elderly subjects between rural city Kyotango and urban city Kyoto: an age‐gender‐matched study. J Clin Biochem Nutr 2019; 65: 125–131. 31592207 10.3164/jcbn.19-26 PMC6769410

[12] Yamamoto Y , Yamamoto T , Miyamoto N *et al*. Oral function and the Oral microbiome in the elderly in the Kyotango area. Dent J (Basel) 2024; 12: 16. 38248224 10.3390/dj12010016 PMC10814942

[13] Naito Y , Yasuda T , Kitae H *et al*. A cross‐sectional study on the relationship between nutrient/food intake and gut microbiota in frailty among older community residents: the Kyotango study. J Clin Biochem Nutr 2024; 75: 161–173. 39345290 10.3164/jcbn.24-93 PMC11425074

[14] Choi L , Liu Z , Matthews CE , Buchowski MS . Validation of accelerometer wear and nonwear time classification algorithm. Med Sci Sports Exerc 2011; 43: 357–364. 20581716 10.1249/MSS.0b013e3181ed61a3 PMC3184184

[15] Choi L , Ward SC , Schnelle JF , Buchowski MS . Assessment of wear/nonwear time classification algorithms for triaxial accelerometer. Med Sci Sports Exerc 2012; 44: 2009–2016. 22525772 10.1249/MSS.0b013e318258cb36 PMC3443532

[16] Ainsworth BE , Haskell WL , Herrmann SD *et al*. Compendium of physical activities: a second update of codes and MET values. Med Sci Sports Exerc 2011; 43: 1575–1581. 21681120 10.1249/MSS.0b013e31821ece12

[17] Aguilar‐Farías N , Brown WJ , Peeters GM . ActiGraph GT3X+ cut‐points for identifying sedentary behaviour in older adults in free‐living environments. J Sci Med Sport 2014; 17: 293–299. 23932934 10.1016/j.jsams.2013.07.002

[18] Sasaki JE , John D , Freedson PS . Validation and comparison of ActiGraph activity monitors. J Sci Med Sport 2011; 14: 411–416. 21616714 10.1016/j.jsams.2011.04.003

[19] Bezuidenhout L , Thurston C , Hagströmer M , Moulaee Conradsson D . Validity of hip and ankle worn Actigraph accelerometers for measuring steps as a function of gait speed during steady state walking and continuous turning. Sensors (Basel) 2021; 21: 3154. 34062943 10.3390/s21093154 PMC8124409

[20] Costa Pereira JPD , Rebouças AS , Prado CM *et al*. Phase angle as a marker of muscle quality: a systematic review and meta‐analysis. Clin Nutr 2024; 43: 308–326. 39549478 10.1016/j.clnu.2024.11.008

[21] Mekary RA , Willett WC , Hu FB , Ding EL . Isotemporal substitution paradigm for physical activity epidemiology and weight change. Am J Epidemiol 2009; 170: 519–527. 19584129 10.1093/aje/kwp163 PMC2733862

[22] Bull FC , Al‐Ansari SS , Biddle S *et al*. World Health Organization 2020 guidelines on physical activity and sedentary behaviour. Br J Sports Med 2020; 54: 1451–1462. 33239350 10.1136/bjsports-2020-102955 PMC7719906

[23] Jefferis BJ , Sartini C , Lee IM *et al*. Adherence to physical activity guidelines in older adults, using objectively measured physical activity in a population‐based study. BMC Public Health 2014; 14: 382. 24745369 10.1186/1471-2458-14-382 PMC4021412

[24] Skender S , Ose J , Chang‐Claude J *et al*. Accelerometry and physical activity questionnaires ‐ a systematic review. BMC Public Health 2016; 16: 515. 27306667 10.1186/s12889-016-3172-0 PMC4910242

[25] Chen S , Chen T , Kishimoto H , Yatsugi H , Kumagai S . Associations of objectively measured patterns of sedentary behavior and physical activity with frailty status screened by the frail scale in Japanese community‐dwelling older adults. J Sports Sci Med 2020; 19: 166–174. 32132840 PMC7039012

[26] Ishihara Y , Ozaki H , Nakagata T *et al*. Association between daily physical activity and locomotive syndrome in community‐dwelling Japanese older adults: a cross‐sectional study. Int J Environ Res Public Health 2022; 19: 8164. 35805823 10.3390/ijerph19138164 PMC9265950

[27] Park H , Park S , Shephard RJ , Aoyagi Y . Yearlong physical activity and sarcopenia in older adults: the Nakanojo study. Eur J Appl Physiol 2010; 109: 953–961. 20336310 10.1007/s00421-010-1424-8

[28] Namba H , Yamada Y , Ishida M , Takase H , Kimura M . Use of a web‐based physical activity record system to analyze behavior in a large population: cross‐sectional study. J Med Internet Res 2015; 17: e74. 25794109 10.2196/jmir.3923 PMC4383835

[29] Abe T , Okuyama K , Hamano T , Takeda M , Isomura M , Nabika T . Hilly environment and physical activity among community‐dwelling older adults in Japan: a cross‐sectional study. BMJ Open 2020; 10: e033338. 10.1136/bmjopen-2019-033338 PMC7170569 32220911

[30] Okuyama K , Abe T , Li X , Toyama Y , Sundquist K , Nabika T . Neighborhood environmental factors and physical activity status among rural older adults in Japan. Int J Environ Res Public Health 2021; 18: 1450. 33557194 10.3390/ijerph18041450 PMC7913898

[31] Ohta R , Yakabe T , Adachi H , Sano C . The association between the frequency of agriculture and control of chronic diseases among regular patients in rural community hospitals: a cross‐sectional study. Cureus 2024; 16: e62849. 39040754 10.7759/cureus.62849 PMC11260665

[32] Yatsugi H , Chen T , Chen S , Liu X , Kishimoto H . The associations between objectively measured physical activity and physical function in community‐dwelling older Japanese men and women. Int J Environ Res Public Health 2021; 19: 369. 35010628 10.3390/ijerph19010369 PMC8744806

[33] Matias CN , Nunes CL , Francisco S *et al*. Phase angle predicts physical function in older adults. Arch Gerontol Geriatr 2020; 90: 104151. 32563736 10.1016/j.archger.2020.104151

[34] Aoyagi Y , Shephard RJ . Habitual physical activity and health in the elderly: the Nakanojo study. Geriatr Gerontol Int 2010; 10: S236–S243. 20590838 10.1111/j.1447-0594.2010.00589.x

[35] Hanibuchi T , Kawachi I , Nakaya T , Hirai H , Kondo K . Neighborhood built environment and physical activity of Japanese older adults: results from the Aichi gerontological evaluation study (AGES). BMC Public Health 2011; 11: 657. 21854598 10.1186/1471-2458-11-657 PMC3170622

[36] Amagasa S , Fukushima N , Kikuchi H , Takamiya T , Oka K , Inoue S . Light and sporadic physical activity overlooked by current guidelines makes older women more active than older men. Int J Behav Nutr Phys Act 2017; 14: 59. 28464833 10.1186/s12966-017-0519-6 PMC5414194

[37] Mañas A , Del Pozo‐Cruz B , Guadalupe‐Grau A *et al*. Reallocating accelerometer‐assessed sedentary time to light or moderate‐ to vigorous‐intensity physical activity reduces frailty levels in older adults: an Isotemporal substitution approach in the TSHA study. J Am Med Dir Assoc 2018; 19: 185.e1–185.e6. 10.1016/j.jamda.2017.11.003 29269096

[38] Chien SY , Wang TH , Tzeng YL , Lu SH , Chang TS . Time allocation to physical activity and sedentary behaviour and its impact on sarcopenia risk: a systematic review and meta‐analysis. J Adv Nurs 2025; 81: 6250–6260. 39936334 10.1111/jan.16781

